# Zexie decoction alleviates hyperlipidemia through modulation of the PPAR signaling pathway and lysophospholipid metabolism

**DOI:** 10.1080/13880209.2026.2687990

**Published:** 2026-06-15

**Authors:** Sen Li, Zhao-Quan Wen, Pan Yan

**Affiliations:** ^a^ Department of Pharmacy, Union Hospital, Tongji Medical College, Huazhong University of Science and Technology, Wuhan, China; ^b^ Department of Chemical Engineering, School of Environmental and Chemical Engineering, Jiangsu Ocean University, Lianyungang, China; ^c^ Department of Pharmacy, The Affiliated Changsha Central Hospital, Hengyang Medical School, University of South China, Changsha, China

**Keywords:** Zexie decoction, hyperlipidemia, serum pharmacochemistry, network pharmacology, targeted lipidomics

## Abstract

**Context:**

Zexie Decoction (ZXD) is a classical traditional Chinese medicine prescription that has long been used for the treatment of hyperlipidemia. However, its material basis and underlying mechanisms remain unclear.

**Objective:**

This research aims to explore the effective compounds and lipid-lowering mechanism of ZXD in the treatment of hyperlipidemia by integrating serum pharmacochemistry, network pharmacology and targeted lipidomics.

**Materials and methods:**

A high-fat diet-induced hyperlipidemia mouse model was established for evaluating the therapeutic efficacy of ZXD. The absorbed components of ZXD in serum were identified using ultra-high performance liquid chromatography quadrupole time-of-flight mass spectrometry (UHPLC-QTOF-MS/MS). Network pharmacology analysis based on *in vivo* absorbed components was subsequently performed to construct molecular networks and predict potential mechanisms. Furthermore, targeted lipidomics was employed to characterize lysophospholipid metabolic changes associated with ZXD treatment for hyperlipidemia.

**Results:**

Pharmacodynamic evaluation demonstrated that ZXD exerted significant lipid-lowering effects on hyperlipidemic mice. Serum pharmacochemistry analysis identified twelve absorbed constituents, including 3 prototype compounds and 9 metabolites. Network pharmacology analysis demonstrated that ZXD primarily modulated lipid metabolism-associated pathways, particularly the peroxisome proliferator-activated receptor (PPAR) signaling pathway and the lipid and atherosclerosis pathways. Molecular docking further revealed strong binding affinities between the major bioactive compounds of ZXD and key targets PPARA and PPARG. In addition, targeted lipidomics identified 36 lysophospholipid biomarkers associated with ZXD-mediated amelioration of hyperlipidemia.

**Discussion and conclusions:**

Integrated analysis indicates that ZXD alleviates hyperlipidemia in association with modulation of the PPAR signaling pathway and lysophospholipid metabolism, highlighting a potential lysophospholipid-PPAR regulatory mechanism.

## Introduction

Hyperlipidemia, a disorder of lipid metabolism, is characterized by increased levels of total cholesterol (TC), triglycerides (TG), and low-density lipoprotein-cholesterol (LDL-C) or a decreased level of high-density lipoprotein-cholesterol (HDL-C) (Wei et al. 2024). It is a major modifiable risk factor for developing atherosclerotic cardiovascular disease (Li et al. 2023). In China, the prevalence of dyslipidemia has reached approximately 40% (Wang et al. 2023). Currently, statins are first-line pharmacological agents for the clinical management of hyperlipidemia; however, long-term statin therapy is often accompanied by adverse effects such as myopathy and rhabdomyolysis (Duan et al. 2024). Therefore, the development of safer and more effective lipid-lowering therapies remains an important clinical need.

Traditional Chinese medicines (TCMs) have the advantage of stable therapeutic efficacy and minor side effects in treating hyperlipidemia (Ren et al. 2023). Zexie Decoction (ZXD), a classic TCM formula, recorded in *Synopsis of the Golden Chamber*, consists of *Alismatis Rhizoma* (ZX, the rhizome of *Alisma orientale (Sam.)* Juzep.) and *Atractylodis Macrocephalae Rhizoma* (BZ, the rhizome of *Atractylodes macrocephala* Koidz.) at a ratio of 5:2 (Wu et al. 2021; Shi et al. 2025). It has been widely used to treat various ailments, such as nonalcoholic fatty liver, atherosclerosis and hyperlipidemia (Wu et al. 2022; Ju et al. 2024). Although some studies have reported the anti-hyperlipidemia effect of ZXD (Xu et al. 2021; Xie et al. 2022; Ju et al. 2024), the complex chemical composition and unclear mechanisms of action have limited its broader clinical application.

In recent years, with continuous innovations in research theories and technologies, methodological frameworks guided by TCM's principles of holistic regulation and syndrome treatment, specifically including serum pharmacochemistry, network pharmacology, and metabolomics, have become a hotspot (Xiong et al. 2022). Serum pharmacochemistry, a well-recognized research method for analyzing and screening active compounds in TCM, can effectively avoid the false-positive or false-negative results caused by the blind separation of chemical components *in vitro* (Liu et al. 2024a). Network pharmacology is an interdisciplinary field that integrates systems biology, bioinformatics, and other disciplines to reveal the molecular interactions of active compounds with therapeutic targets (Liu et al. 2024b; Pang et al. 2025). Lipidomics, a subfield of metabolomics, is often used to identify and reveal the lipid biomarkers associated with diseases or treatments (Yan et al. 2022). The integration of these in-silico approaches and experimental studies will provide a powerful tool to elucidate the material basis and mechanisms of action for TCM.

In this study, an integrated strategy combining serum pharmacochemistry, network pharmacology, and targeted lipidomics was used to systematically investigate the material basis and lipid-lowering mechanism of ZXD in the treatment of hyperlipidemia. To our knowledge, this integrated strategy has been applied for the first time in ZXD-related research to explore the ameliorative mechanism of ZXD in hyperlipidemia from the perspectives of active compounds, molecular interaction networks, and lysophospholipids.

## Materials and methods

### Chemicals and reagents

Acetonitrile (HPLC grade) was purchased from Fisher Scientific (Fair Lawn, NJ, United States). Ultrapure water was purchased from Wahaha Group (Hangzhou, China). Formic acid (HPLC grade, FA) and carboxymethylcellulose sodium (CMC-Na) was purchased from Sinopharm Chemical Reagent Co., Ltd (Shanghai, China). Simvastatin was obtained from Merck Pharmaceutical Co., Ltd. (Hangzhou, China). The reference standards of lysophospholipids (Lyso-PC/16:0, 16:0/Lyso-PC, Lyso-PE/16:0, 16:0/Lyso-PE, Lyso-PS/18:1, 18:1/Lyso- PS) were obtained from Avanti Polar Lipids Inc (Alabaster, AL).

### Preparation of ZXD extracts

ZX and BZ were purchased from the TCM dispensary of Union Hospital (Wuhan, China) and were authenticated by Prof. Shuna Jin (Hubei University of Chinese Medicine). The voucher specimens (no. ZX20240801-01 and BZ20240801-01) were deposited at the herbarium of the Pharmacy Laboratory, Union Hospital, Tongji Medical College, Huazhong University of Science and Technology.

The samples of ZXD were prepared by mixing ZX and BZ in a 5:2 ratio (*w/w*). The mixture was decocted three times with ultra-pure water (1:10, *w/v*) for 2 h each. The combined decoctions were filtered and concentrated under reduced pressure, followed by vacuum drying at 60 °C for 48 h. The extraction yield was 12.3%. The dried extracts were dissolved in 0.5% CMC-Na solution to a final concentration equivalent to 2 g/mL of raw herbal material. To detect the prototype compounds of ZXD, 50 mg of dried extracts were diluted in 10 mL of 50% ethanol, centrifuged at 12000 r/min for 15 min and filtered with a 0.22 μm filter.

### Animals and treatment

Twenty-four 6-week-old male Kunming (KM) mice (18-22 g) were kept for 3 days under standard conditions with a 12 h light/dark cycle. Then, these mice were randomly divided into two groups: the normal control (NC) group (*n* = 6) receiving standard chow and the hyperlipidemia model group (*n* = 18) receiving a high-fat diet. Based on our previous studies, the formulation of the high-fat diet was 78.8% commercial diet, 10% egg yolk, 10% lard, 1% cholesterol, and 0.2% cholate (Li et al. 2016; Yan et al. 2022). After 4 weeks, blood was collected from the orbital vein of high-fat diet-fed mice to measure blood lipid parameters. The significant changes in plasma lipid levels, together with the manifestation of phlegm-dampness retention symptoms, such as obesity, lassitude, and loose and sticky stools (Chen et al. 2023), indicated that the hyperlipidemia model had been successfully established. Subsequently, the hyperlipidemia model group was randomly divided into 3 groups (6 mice per group): high-fat diet (HFD) group (*n* = 6), simvastatin (SIM) group (*n* = 6) and ZXD group (*n* = 6). No animals were excluded after randomization. According to previous study and literature, the oral gavage doses of simvastatin and ZXD extracts were 20 mg/kg/d and 20 g/kg/d, respectively (Song et al. 2014; Yan et al. 2022). Meanwhile, the NC and HFD groups were intragastrically given 0.5% CMC-Na. The treatment lasted for 4 weeks, with all groups maintaining the same diets as described previously. At the end of treatment, all groups were fasted for 12 h. The blood samples were collected in EDTA-2K-containing EP tubes under anesthesia, followed by centrifugation at 3000 *g* for 10 min at 4 °C to obtain plasma. The plasma samples were stored at −80 °C for biochemistry analysis and targeted lipidomics. The liver was immediately dissected, rinsed with cold phosphate-buffered saline (PBS), and a portion was fixed in 4% paraformaldehyde solution for histopathological examination.

To investigate the serum pharmacochemistry of ZXD, another cohort of male KM mice (*n* = 30) was acclimated for 3 days under standard laboratory conditions. Fifteen mice received ZXD extracts (20 g/kg/d) *via* oral gavage, while the remaining 15 (blank group) were administered an equal volume of 0.5% CMC-Na vehicle, for 3 consecutive days. The blood samples were collected from the ocular orbit at 15 and 30 min, 1, 2, and 4 h after the last administration (3 mice per time point) (Li et al. 2025). The blood samples were incubated at room temperature for 30 min, and then centrifuged at 3000 *g* and 4 °C for 10 min to separate the serum. Equal amounts of serum at different time points were mixed to obtain the pooled serum sample. The mixed serum samples were stored at −80 °C until serum pharmacochemistry analysis.

### Biochemistry analysis

Plasma biochemical parameters, including TG, TC, HDL-C and LDL-C, were determined using commercial assay kits (Nanjing Jiancheng Bioengineering Institute, Nanjing, China) according to the manufacturer's instructions.

### Histological examinations

Frozen liver sections were embedded in Optimum Cutting Temperature (OCT) compound and subsequently stained with Oil Red O to visualize lipid droplets. Lipid accumulation in the liver tissues was then observed and assessed under a light microscope.

### Serum pharmacochemistry analysis

Protein precipitation was performed on 1 mL of pooled serum sample using 5 volumes of methanol. The mixture was thoroughly vortexed for 5 min, followed by centrifugation at 17000 × g and 4 °C for 10 min. The resulting supernatant was evaporated to dryness under a gentle nitrogen stream, and then reconstituted in 300 μL of acetonitrile-water (1:1, v/v) (Yan et al. 2022). Prior to LC-MS/MS analysis, all samples were filtered through 0.22 μm membrane filters.

Waters H-class UPLC system (Waters Corporation, Milford, MA, United States) was used for the Chromatographic. Separation was carried out on a Welch Ultimate UPLC AQ-C_18_ (2.1 × 100 mm, 1.8 μm). The elution system was acetonitrile (A) and 0.1% aqueous formic acid solution (B), while linear gradient elution optimization was performed as follows: 0 min, 100% A; 15.0 min, 8% A; 22.0 min, 45% A; 28.0 min, 75% A; 33.0 min, 95% A; 37.0 min, 95% A. The flow rate was set at 0.3 mL/min, the column temperature was kept at 35 °C, and the sample injection volume was 3.0 μL.

Mass spectrometric analysis was performed on a Triple TOF™ 4600 system with a Duo Spray source (AB SCIEX, Foster City, CA, USA) in positive and negative electrospray ionization (ESI) mode. The MS conditions were as follows: ion spray voltage, 4.5 kV; the ion source temperature, 550 °C; curtain gas, 25 psi; nebulizer gas (GS1), 50 psi; heater gas (GS 2), 50 psi; declustering potential (DP), 100 V. The mass ranges were set at *m/z* 50-1700 for TOF MS scan, 50-1250 for TOF MS/MS experiments. The collision energy was set at ± 40 eV and the collision energy spread was 20 eV for MS/MS experiments.

Data acquisition was performed using Analyst TF 1.7.1 software, and the data were processed with PeakView 1.2. The prototype constituents of ZXD were tentatively identified by matching spectra against in-house database, commercial spectral library, and relevant literature. Subsequently, the absorbed prototype compounds and metabolites of ZXD were characterized by comparative analysis of the ZXD extracts, dosed serum, and blank serum samples.

### Network pharmacology analysis

#### Prediction of the target proteins

The prototypes and metabolites absorbed into the blood after administration were more likely to be pharmacodynamic constituents of TCMs (Tang et al. 2022; Liu et al. 2024a). Based on the serum pharmacochemistry results, the identified prototype compounds and phase I metabolites were used to construct the chemical information database of ZXD for subsequent network pharmacology research (Yu et al. 2019). The component-related targets were obtained from the following databases: ETCM (Xu et al. 2019), HERB (Gao et al. 2025), PharmMapper (Liu et al. 2010), and SwissTargetPrediction (Daina et al. 2019). The disease-related targets were retrieved with the keyword “hyperlipidemia” from the GeneCards, TTD (Zhang et al. 2026), and OMIM databases.

These targets were standardized to official gene symbols using the UniProt database. Then, the intersection between component-related and disease-related targets was defined as the potential targets for the treatment of hyperlipidemia with ZXD.

#### Network construction and analysis

A compound-target-disease (C-T-D) network was constructed, where each node represents active compounds (prototypes and metabolites of ZXD), potential targets, and hyperlipidemia, respectively. The protein-protein interaction (PPI) network of potential targets was constructed using the STRING database with default parameters. Both the C-T-D and PPI networks were visualized by Cytoscape. Hub targets within the PPI network were subsequently identified using the CytoHubba plugin.

#### Functional enrichment and pathway analysis

The Gene Ontology (GO) and Kyoto Encyclopedia of Genes and Genomes (KEGG) analyses were conducted using the DAVID database (Sherman et al. 2022) and visualized using the Weishengxin online platform.

### Molecular docking

Molecular docking was performed using CB-Dock2. This tool can automate cavity detection and the docking process (Liu et al. 2022b). The binding energy values for the receptor-ligand complex were evaluated using Vina score. The docking conformation was visualized and interpreted by PyMOL and LigPlot^+^ software.

### Targeted lipidomics analysis

Targeted lipidomics analysis was performed using plasma samples from the NC, HFD, and ZXD groups, with six biological replicates included in each group. Plasma (100 μL) was mixed with 500 μL of acetonitrile and vortexed for 2 min. The samples were sonicated for 15 min in ice-water bath, and centrifuged at 14000 × g at 4 °C for 15 min. 500 μL of supernatant was dried under nitrogen gas. The residue was then dissolved in 100 μL of 50% aqueous methanol (containing curcumin internal standard). The samples were filtered with a 0.22 μm microfilters prior to further LC-MS/MS analysis.

According to the previous study (Li et al. 2016; Mengxiang et al. 2024). the liquid chromatography coupled with tandem mass spectrometer (LC-MS/MS) conditions were described as follows. The LC-MS/MS analyses were performed on an ultra-high-performance liquid chromatography (UHPLC) system (Waters, US), equipped with a Waters C_18_ column (2.1 mm × 100 mm, 1.7 μm) at a flow rate of 0.3 mL/min. The column oven was set at 40 °C. The mobile phase consisted of 0.1% formic acid aqueous solution (A) and acetonitrile (B). The analysis was carried out with an elution gradient as follows: 0-7 min, 10%-55% B; 7-26 min, 55% B; 26-35 min, 55%-75% B; 35-46 min, 75%-95% B; 46-54 min, 95% B; 54-55 min, 95%-10% B; 55-60 min, 10% B. The injection volume was 5 μL.

The quadrupole time of flight (Q-TOF) system (Xevo G2-XS, Waters, US) was used to acquire MS/MS spectra and operated in positive ion mode. The MS conditions were as follows: ion spray voltage, 3000 V; desolvation temperature, 500 °C; cone gas flow, 50 L/h; desolvation gas flow, 600 L/h; cone voltage, 20 V; The mass range was established at *m/z* values of 100 to 1200 for a TOF-MS scan and *m/z* values of 50-1200 for a TOF-MS/MS scan. Data processing and analysis were facilitated using MassLynx software.

### Statistical analysis

Statistical analyses were performed using GraphPad Prism 9 Software (GraphPad Instruments, USA). A one-way analysis of variance (ANOVA) was used for normal distribution, while a nonparametric rank sum test was used for non‑normal distribution. The *P*-value < 0.05 was considered statistically significant.

## Results

### Hypolipidemic activity of ZXD in hyperlipidemia

**Figure 1.**
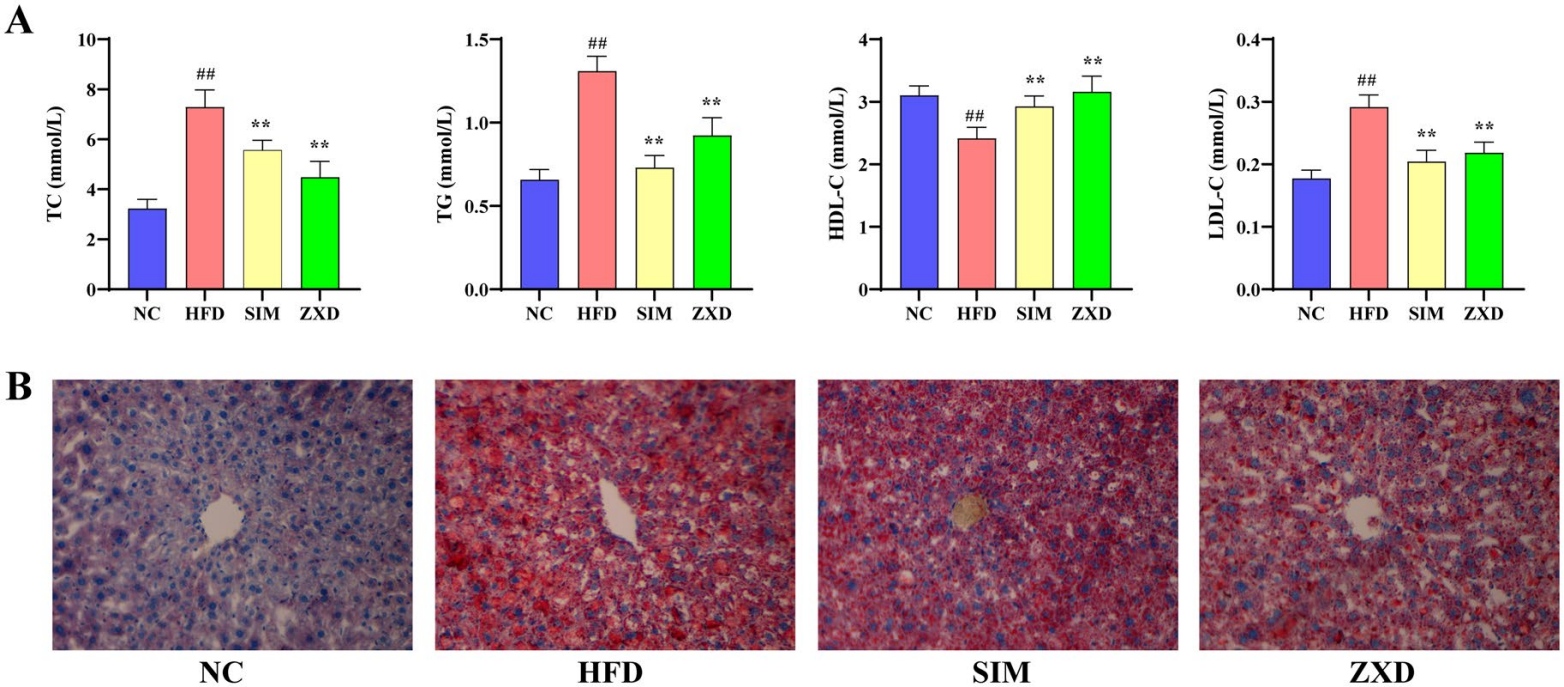
The pharmacodynamic results of ZXD against hyperlipidemia. (A) Effects of ZXD on plasma lipid levels in hyperlipidemic mice, (B) Oil red O staining section of liver. Data are presented as mean ± SD. ^#^*p* < 0.05, ^##^*p* < 0.01 versus NC group; *p < 0.05, **p < 0.01 versus HFD group.

At the end of hyperlipidemia modeling, baseline levels of biochemical parameters differed significantly between the high-fat diet group and the normal control group, confirming successful establishment of the hyperlipidemia model. In contrast, no significant differences were observed among the HFD, SIM, and ZXD subgroups before intervention, indicating good baseline comparability among these subgroups (Figure S1). After the intervention, biochemical analysis revealed that compared to the NC group, the high-fat diet significantly elevated plasma levels of TC, TG, and LDL-C (all *p* < 0.01), and significantly reduced plasma HDL-C levels (*p* < 0.01) in the HFD group (Figure 1(A)). After four weeks of treatment with ZXD or simvastatin, these biochemical parameters were markedly reversed, exhibiting a trend toward normalization in hyperlipidemic mice (all *p* < 0.01). These results indicated that ZXD effectively ameliorates high-fat diet-induced dyslipidemia.

The results of the Oil Red-O staining of each group were shown in Figure 1(B). Only a small number of lipid droplets were observed in the liver tissue sections of the NC group. In contrast, abundant lipid droplets were detected in the HFD group, indicating that high-fat diet intake promoted hepatic lipid accumulation in mice. After four weeks of treatment, both ZXD and simvastatin remarkably reduced the number and size of lipid droplets of livers in hyperlipidemic mice. These results suggested that ZXD effectively attenuated hepatic lipid accumulation and improved liver lipid metabolism in hyperlipidemia.

### Identification of ZXD-absorbed components in vivo

A total of 53 prototype compounds of ZXD were tentatively identified by comparison with an in-house database (Li et al. 2016; Wang et al. 2021; Yan et al. 2021), the Natural Products HR-MS/MS Spectral Library 1.0 (Shanghai Standard Technology Co., Ltd.) and published literature (Sun et al. 2017; Xin et al. 2017; ZHANG et al. 2019; Liu et al. 2022a). The results were listed in Table S1, and the base peak chromatogram (BPC) was shown in Figure S2. Following a comprehensive analysis of chemical constituents present in the ZXD extracts and dosed serum samples (Figure S3), a total of 12 compounds absorbed into the bloodstream were identified, including 3 prototype compounds and 9 metabolites. The detailed information of the identified compounds was summarized in Table 1. For instance, M9 was identified as a metabolite derived *via* hydroxylation of alisol B. Based on MS/MS fragmentation rules, alisol B had characteristic ions at *m/z* 383 and 339. The M9 had characteristic ions at *m/z* 381 and 337, indicating that the oxidation reaction occurred on the parent nucleus. Therefore, according to the potential metabolic pathways and characteristic fragment ions, M9 was tentatively identified as 16-hydroxy-Alisol B (Wang et al. 2021; Yan et al. 2021).

**Table 1.** Identification of prototype compounds and metabolites in mouse serum after administration of ZXD by UHPLC-MS/MS.

No.	RT (min)	Adduct	Experimental (*m/z*)	Calculated (*m/z*)	Error (ppm)	Formula	MS/MS Fragments	Identification
P1	17.38	[M + FA−H]^−^	493.2308	493.2300	1.6	C_21_H_36_O_10_	493.2337, 447.229, 285.1754	Atractyloside A
P2	26.89	[M + H]^+^	529.3519	529.3524	−0.9	C_32_H_48_O_6_	529.3519, 469.3298, 451.3112, 433.3153	Alisol C 23-acetate
P3	29.98	[M−H_2_O + H]^+^	455.3513	455.3520	−1.5	C_30_H_48_O_4_	437.3320, 383.2990, 365.2948, 205.1585, 145.1029	Alisol B
M1	13.34	[M−H]^−^	357.0810	357.0827	−4.8	C_15_H_18_O_10_	357.0804, 181.0500, 137.0616, 113.0245	Caffeicacid + hydrogenation + glucuronidation
M2	19.31	[M−H]^−^	258.9915	258.9918	−1.2	C_9_H_8_O_7_S	179.0349, 135.0455	Caffeic acid + sulfation
M3	19.79	[M−H]^−^	206.0834	206.0823	5.3	C_11_H_13_NO_3_	206.0816, 164.0734, 147.0463, 125.8429	Ferulic acid amide + methylation
M4	19.82	[M−H]^−^	275.0231	275.0231	0.0	C_10_H_12_O_7_S	195.0640, 177.0535	Ferulic acid + hydrogenation + sulfation
M5	20.23	[M−H]^−^	258.9918	258.9918	0.0	C_9_H_8_O_7_S	258.9915, 179.0349, 135.0449	Caffeic acid + sulfation
M6	20.24	[M−H]^−^	245.0131	245.0125	2.3	C_9_H_10_O_6_S	245.0115, 165.0553, 121.0654, 119.0497	Caffeicacid + hydrogenation + dihydroxylation + sulfation
M7	20.45	[M−H]^−^	273.0072	273.0074	−0.7	C_10_H_10_O_7_S	193.0524, 178.0282, 134.0364	Ferulic acid + sulfation
M8	24.41	[M + H]^+^	219.1373	219.1380	−3.2	C_14_H_18_O_2_	201.1354, 191.1385, 159.0780, 131.0844, 121.0625	11-demethylation-atractylenolide II
M9	27.00	[M + H]^+^	489.3556	489.3575	−3.9	C_30_H_48_O_5_	489.3496, 381.2888, 363.2715, 337.2543, 217.1611	16-hydroxy-alisol B

Notes: P, prototype compound; M, metabolite.

### Network pharmacology analysis

#### Prediction of target proteins

**Figure 2.**
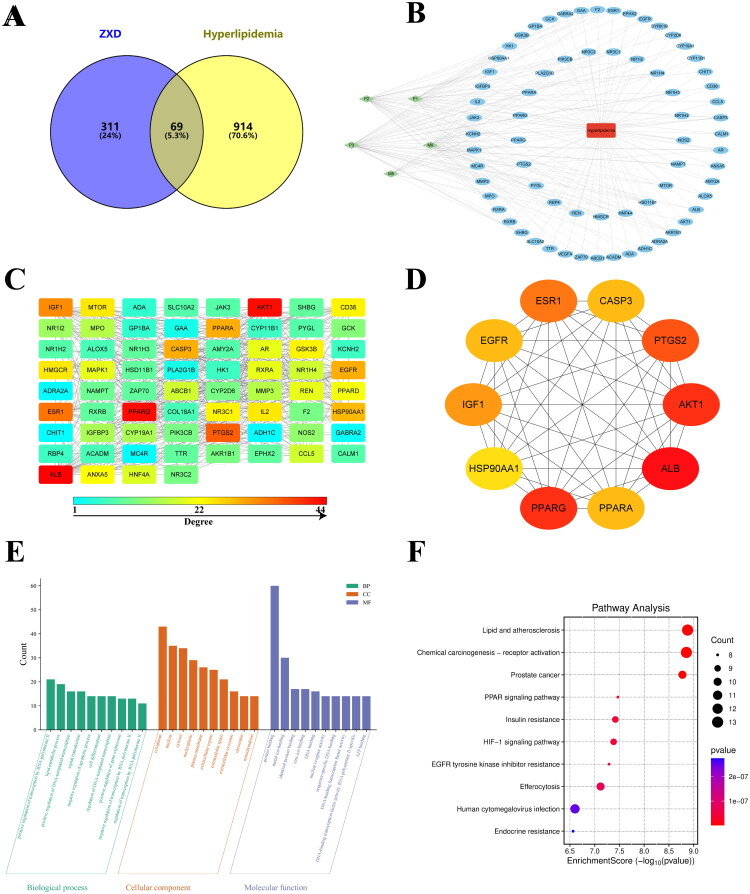
Network pharmacology results of ZXD against hyperlipidemia. (A) Venn diagram of the intersection targets between ZXD and hyperlipidemia, (B) C-T-D network, (C) PPI network, (D) Map of the hub targets screened from PPI by CytoHubba, (E) Triple plots of the top 10 enrichment results of each of the GO function BP, CC and MF, (F) Bubble chart of KEGG pathways.

Blood-absorbed prototypes and metabolites are potential active compounds underlying the therapeutic effects of TCM. In this study, considering that prototype compounds and phase I metabolites have definite chemical structures, five serum-absorbed constituents, including 3 prototype compounds and 2 phase I metabolites, were selected for network pharmacology analysis. A total of 380 ZXD-related targets were predicted from ETCM, HERB, PharmMapper and SwissTargetPrediction. Subsequently, a total of 983 hyperlipidemia-related targets were retrieved using the keyword 'Hyperlipidemia' from GeneCards, TTD and OMIM. Between ZXD-related and hyperlipidemia-related targets, 69 intersection targets were identified as potential targets for ZXD against hyperlipidemia (Figure 2(A)).

#### C-T-D and PPI network

As shown in Figure 2(B), the C-T-D network illustrates the relationships among the 5 active compounds (3 prototypes and 2 metabolites of ZXD), 69 intersection targets and hyperlipidemia. In the C-T-D network, the green diamonds represent the prototypes and metabolites absorbed into serum, the blue ellipses represent the potential therapeutic targets of ZXD for hyperlipidemia, and the red rectangle represents the hyperlipidemia. The importance of a node is proportional to its degree, which serves as a measure of the number of edges it possesses. Based on their degree values, alisol B (degree = 39) was identified as the pivotal compound, followed by alisol C 23-acetate (degree = 34), 16-hydroxy-alisol B (degree = 26), 11-demethylation-atractylenolide II (degree = 26), and atractyloside A (degree = 22). This order suggested that the triterpenoids from *Alismatis Rhizoma* were likely the main active constituents responsible for the lipid-lowering effect of ZXD.

By importing the 69 potential targets into the STRING database, a PPI network was generated. The target DYRK1B was excluded because it did not interact with any of the other targets. The PPI network comprised 68 nodes and 514 edges, where red nodes denote targets with a higher degree of interaction (Figure 2(C)). In the PPI network, hub targets were identified using the CytoHubba plugin. The top 10 targets involved in the lipid-lowering effects of ZXD were as follows: peroxisome proliferator-activated receptor gamma (PPARG), peroxisome proliferator-activated receptor alpha (PPARA), albumin (ALB), RAC-alpha serine/threonine-protein kinase (AKT1), prostaglandin G/H synthase 2 (PTGS2), caspase-3 (CASP3), estrogen receptor (ESR1), epidermal growth factor receptor (EGFR), insulin-like growth factor 1 (IGF1), and heat shock protein HSP 90-alpha (HSP90AA1). Hub targets were depicted in Figure 2(D) and detailed in Table S2.

#### GO and KEGG enrichment analyses

In GO analysis, the top 10 enriched terms for biological process (BP), cellular component (CC), and molecular function (MF) were shown in Figure 2(E). Among them, BP-related items predominantly included positive regulation of transcription by RNA polymerase II, lipid metabolic process and positive regulation of DNA-templated transcription. The CC-related items were primarily associated with cytoplasm, nucleus and cytosol. Additionally, the MF-related items were mainly linked to protein binding, metal ion binding and identical protein binding. As shown in Figure 2(F), KEGG enrichment analysis indicated that the pathways significantly affected by ZXD included lipid and atherosclerosis, the PPAR signaling pathway, and insulin resistance. These findings were partly consistent with and correlated to our previous study on the hypolipidemic mechanism of *Alismatis Rhizoma* (Yan et al. 2022). Based on the KEGG enrichment results and our previous findings, the PPAR signaling pathway was identified as the key pathway for ZXD in treating hyperlipidemia, with a detailed pathway diagram provided in Figure S4.

### Molecular docking

**Figure 3.**
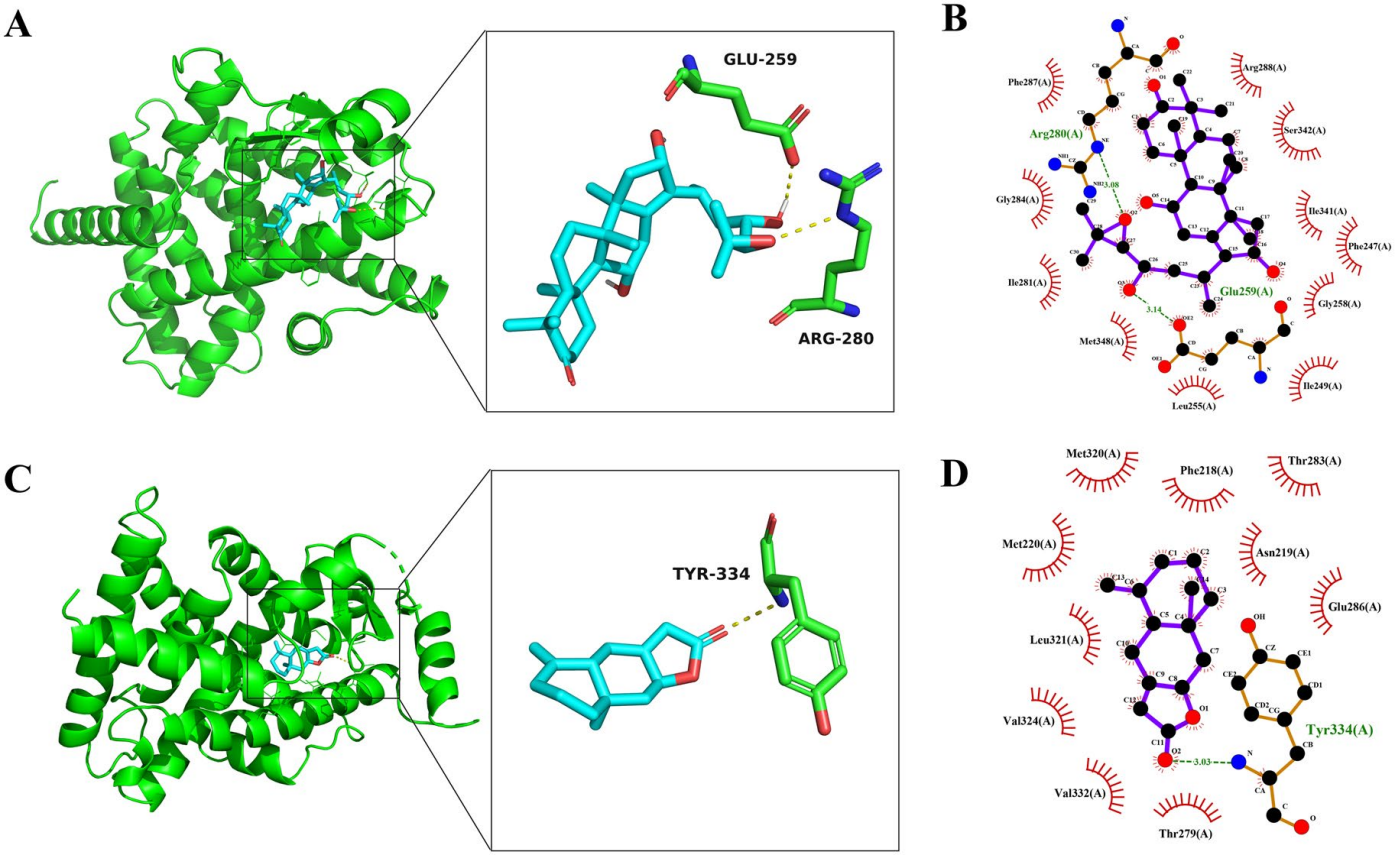
Molecular docking results. (A) The three-dimensional binding pattern between PPARG and 16-hydroxy-alisol B, (B) the two-dimensional interaction map between PPARG and 16-hydroxy-alisol B, (C) The three-dimensional binding pattern between PPARA and 11-demethylation-atractylenolide II, (D) The two-dimensional interaction map between PPARA and 11-demethylation-atractylenolide II.

The hub targets PPARA and PPARG, identified from the PPI network, were selected for molecular docking to verify ZXD's action on the PPAR signaling pathway. The five serum-absorbed compounds were docked separately with PPARA (PDB ID: 6LXC) and PPARG (PDB ID: 6TSG). Based on widely used empirical criteria, vina scores greater than −5 kcal/mol, between −5 and −7 kcal/mol, and less than −7 kcal/mol are generally considered to indicate weak, moderate, and strong binding affinity, respectively (Narayanan and Nazarenko 2025). According to these criteria, the identified active compounds showed moderate to strong binding affinity for PPARA and PPARG (Table S3). In addition, the co-crystallized ligands T4A and EWR were redocked into their corresponding protein cavities as positive controls. The docking scores for the PPARG-T4A and PPARA-EWR complexes were −6.9 kcal/mol and −7.8 kcal/mol, respectively (Figure S5). Interestingly, the active compounds with the highest docking scores (lowest binding energies) against PPARG and PPARA were derived from *Alismatis Rhizoma* and *Atractylodis Macrocephalae Rhizoma*, respectively, and their binding energies were lower than those of the corresponding co-crystallized ligands. This finding suggested a synergistic effect of these two medicinal herbs on lipid-lowering efficacy. For PPARG, 16-hydroxy-alisol B exhibited a binding energy of −7.7 kcal/mol, and its binding was stabilized by hydrogen bonds with the amino acid residues ARG-280 and GLU-259 (Figure 3(A,B)). PPARA and 11-demethylation-atractylenolide II bound to TYR-334 *via* a hydrogen bond, with a binding energy of −8.5 kcal/mol (Figure 3(C,D)).

### Targeted lipidomics

#### Lipidomics data analysis

GO and KEGG enrichment analysis from network pharmacology (Figure 2(E,F)) indicated that the ameliorative mechanism of ZXD against hyperlipidemia was associated with lipid metabolism. Our previous study revealed that the pathogenesis and treatment of hyperlipidemia were closely related to lysophospholipid metabolism (Li et al. 2016; Shi et al. 2019; Mengxiang et al. 2024). Furthermore, lysophospholipid is closely associated with the PPAR signaling pathway (Tsukahara et al. 2017). Therefore, targeted lipidomics was employed to further elucidate the hypolipidemic mechanism of ZXD. In the study, a total of 216 lysophospholipids were identified by matching in-house database (Li et al. 2016; Chen et al. 2017; Shi et al. 2019; Mengxiang et al. 2024).

**Figure 4.**
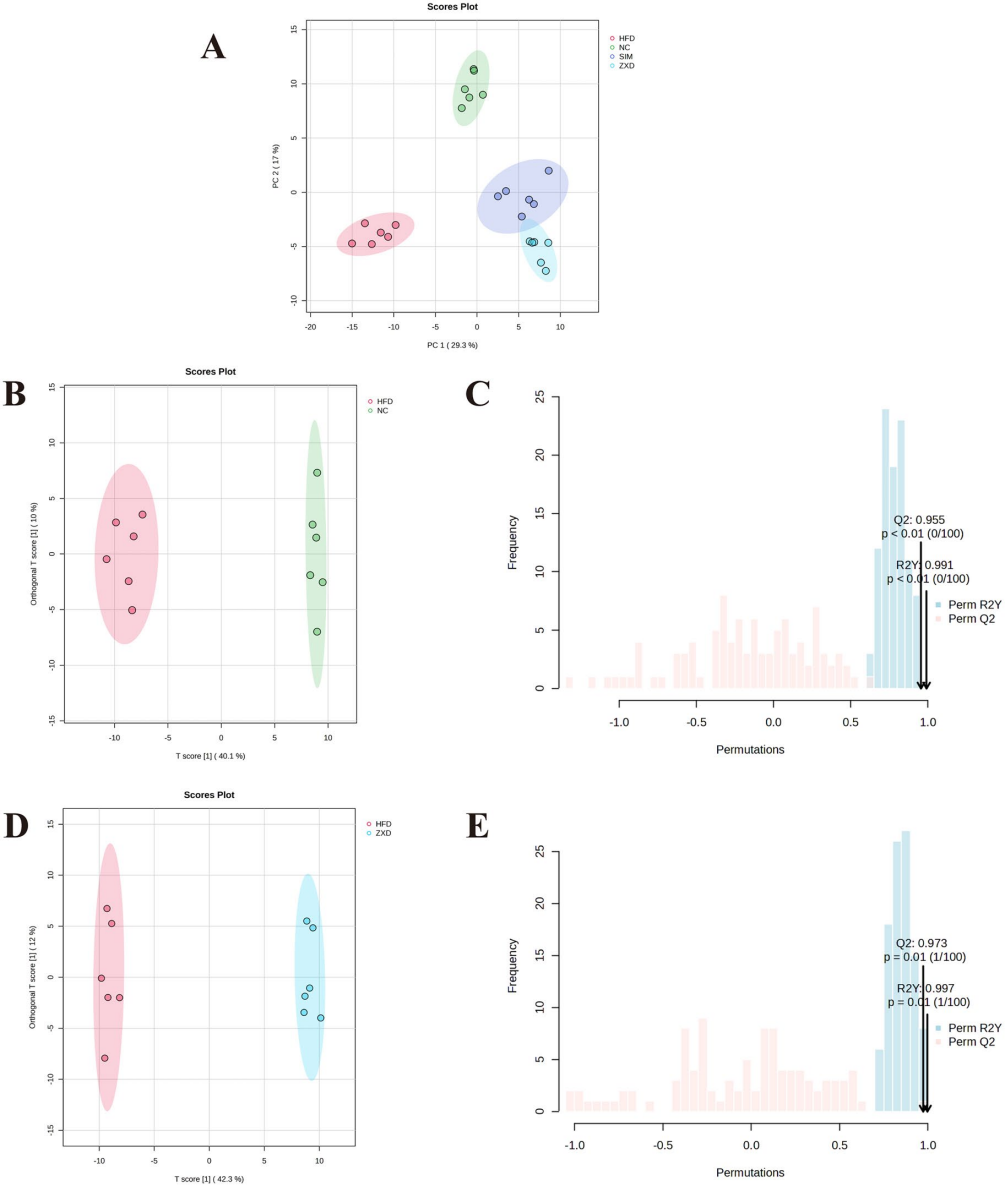
PCA and OPLS-DA plots from lipidomics analysis. (A) PCA score plots of the NC, HFD, SIM, and ZXD groups; (B, C) OPLS-DA score plot and permutation test validation plot for NC vs. HFD; (D, E) OPLS-DA score plot and permutation test validation plot for HFD vs. ZXD.

Principal component analysis (PCA), an unsupervised analytical method, was employed to assess metabolic differences between the sample groups. The PCA plot of the lipidomics data (Figure 4(A)) revealed a clear separation between the NC and HFD groups, indicating that the high-fat diet induced a disorder in lysophospholipid metabolism in hyperlipidemic mice. Meanwhile, the SIM and ZXD groups showed partial overlap and a tendency to cluster closer to the NC group. These results indicated that ZXD had a positive effect in the treatment of hyperlipidemia by improving lysophospholipid metabolism disorders. Orthogonal partial least squares-discriminant analysis (OPLS-DA), a supervised analytical model, was performed to distinguish the metabolic variations and find potential lipid markers. The OPLS-DA scores (Figure 4(B,D)) showed that there were significant differences between the NC group and the HFD group (R^2^Y= 0.972 and Q^2^= 0.939), and between the HFD group and the ZXD group (R^2^Y= 0.992 and Q^2^= 0.963). Subsequently, OPLS-DA models were validated by permutations tests (*n* = 100) to minimize the risk of over-fitting. As shown in Figure 4(C,E), all Q_2_ of the permutation were higher than 0.9 (*p* < 0.05), indicating that there was no over-fitting phenomenon in the OPLS-DA model.

#### Identification of the biomarkers

**Figure 5.**
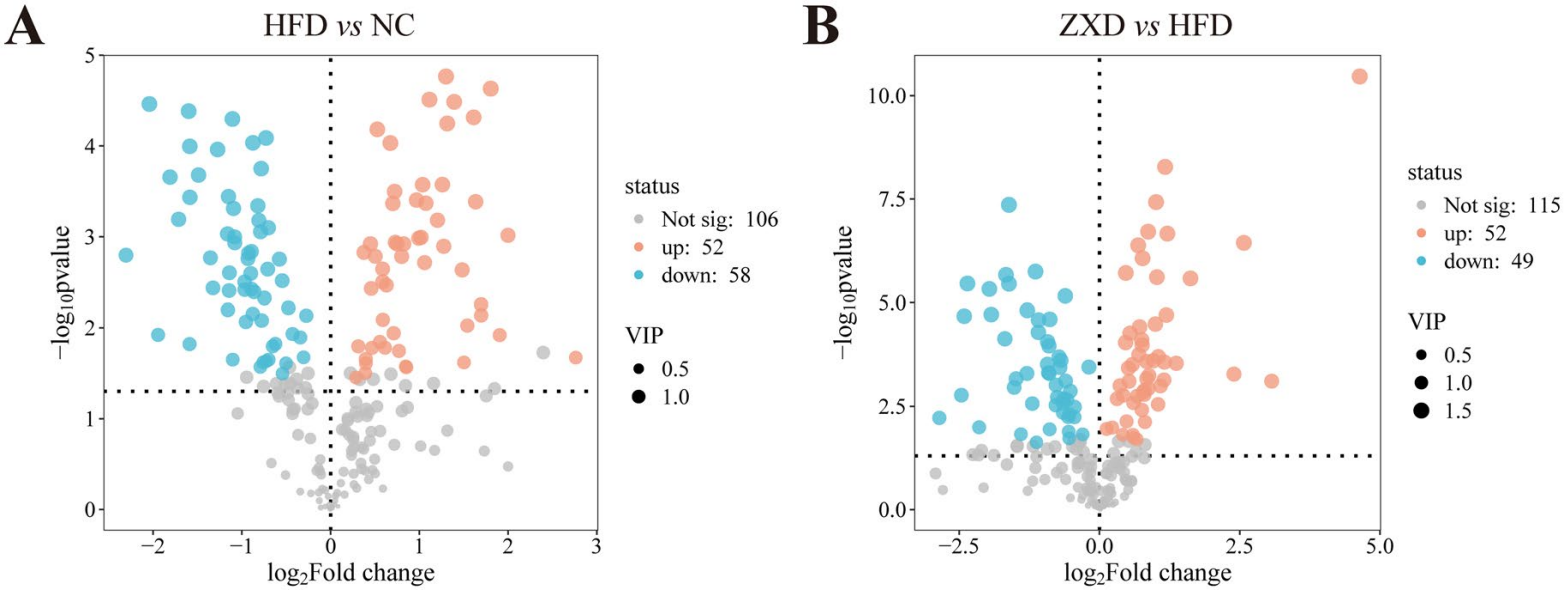
The volcano plot of differentially expressed lysophospholipids. (A) HFD *vs.* NC, (B) ZXD *vs.* HFD.

**Figure 6.**
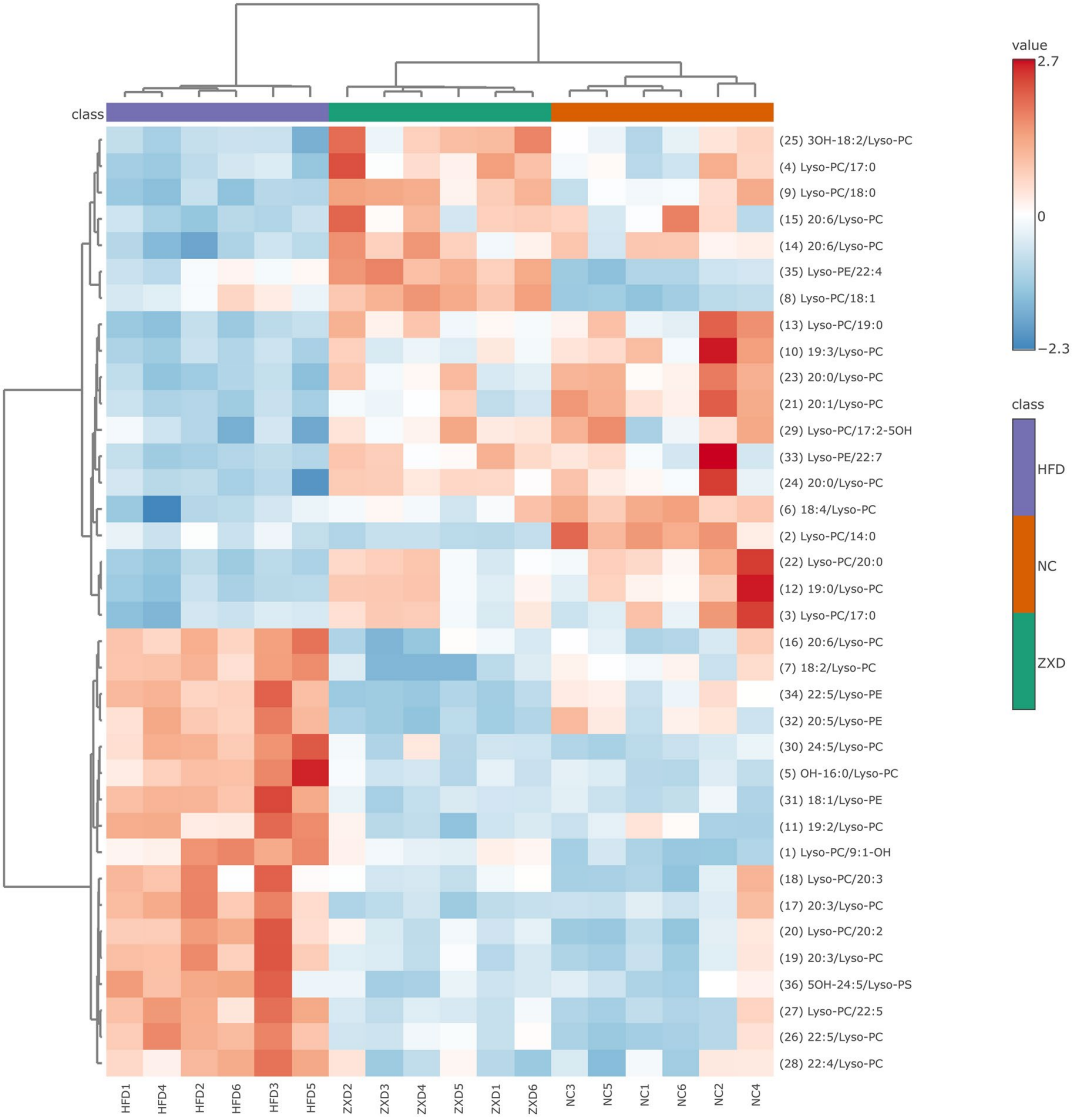
The heatmaps of the differential lipid abundance among all groups. Red for higher level and blue for lower level.

Based on the OPLS-DA models and Student's t-test, the variables with VIP > 1 and *P*-value <0.05 were screened as potential differential lipids to distinguish the two groups. Between the NC and HFD groups, 110 differential lipids were identified, (HFD *vs* NC: 58 down-regulated and 52 up-regulated lysophospholipids). Similarly, 101 differential lipids were obtained from the HFD and ZXD groups (ZXD *vs* HFD: 49 down-regulated and 52 up-regulated lysophospholipids) (Figure 5). A total of 36 overlapping differential lysophospholipids were identified as potential biomarkers for ZXD in the treatment of hyperlipidemia, including 30 lysophosphatidylcholines (Lyso-PCs), 5 lysophosphatidylethanolamines (Lyso-PEs), and 1 lysophosphatidylserine (Lyso-PS). The changing trend of biomarkers between groups was shown in Table 2. The heatmaps was constructed to visualize the relative content of biomarkers among the groups. As shown in Figure 6, the color of the NC group was distinct from the HFD group, while the ZXD group closely resembled the NC group, indicating that ZXD treatment effectively reversed the alterations in these lysophospholipids.

**Table 2.** Differential lysophospholipids characterized in plasma and their change trends.

No	Lipids	C atoms^a^	HFD vs NC			ZXD vs HFD		
			VIP	FC	Trend	VIP	FC	Trend
1	Lyso-PC/9:1-OH	9	1.53	3.06	↑**	1.16	0.64	↓**
2	Lyso-PC/14:0	14	1.40	0.54	↓**	1.04	0.81	↓**
3^#^	Lyso-PC/17:0	17	1.03	0.60	↓*	1.21	1.52	↑**
4^#^	Lyso-PC/17:0	17	1.01	0.81	↓*	1.29	1.44	↑**
5	OH-16:0/Lyso-PC	16	1.36	3.10	↑**	1.29	0.36	↓**
6	18:4/Lyso-PC	18	1.41	0.60	↓**	1.04	1.33	↑*
7	18:2/Lyso-PC	18	1.31	1.68	↑**	1.42	0.19	↓**
8	Lyso-PC/18:1	18	1.36	2.10	↑**	1.34	1.63	↑**
9	Lyso-PC/18:0	18	1.24	0.45	↓**	1.46	3.08	↑**
10	19:3/Lyso-PC	19	1.31	0.58	↓**	1.24	1.34	↑**
11	19:2/Lyso-PC	19	1.28	2.08	↑**	1.30	0.41	↓**
12^#^	19:0/Lyso-PC	19	1.21	0.40	↓**	1.34	2.23	↑**
13^#^	Lyso-PC/19:0	19	1.24	0.61	↓**	1.34	1.50	↑**
14^#^	20:6/Lyso-PC	20	1.33	0.57	↓**	1.35	1.95	↑**
15^#^	20:6/Lyso-PC	20	1.06	0.65	↓*	1.25	1.74	↑**
16^#^	20:6/Lyso-PC	20	1.27	1.42	↑**	1.35	0.62	↓**
17	20:3/Lyso-PC	20	1.30	2.42	↑**	1.46	0.20	↓**
18	Lyso-PC/20:3	20	1.13	1.64	↑*	1.14	0.68	↓**
19	20:3/Lyso-PC	20	1.39	2.05	↑**	1.37	0.53	↓**
20	Lyso-PC/20:2	20	1.37	1.96	↑**	1.31	0.61	↓**
21	20:1/Lyso-PC	20	1.43	0.33	↓**	1.13	1.76	↑**
22^#^	Lyso-PC/20:0	20	1.32	0.30	↓**	1.34	2.58	↑**
23^#^	20:0/Lyso-PC	20	1.42	0.46	↓**	1.27	1.71	↑**
24^#^	20:0/Lyso-PC	20	1.16	0.72	↓**	1.39	1.38	↑**
25	3OH-18:2/Lyso-PC	18	1.08	0.74	↓*	1.34	1.79	↑**
26^#^	22:5/Lyso-PC	22	1.45	2.49	↑**	1.43	0.54	↓**
27^#^	Lyso-PC/22:5	22	1.39	2.39	↑**	1.42	0.47	↓**
28	22:4/Lyso-PC	22	1.21	1.37	↑**	1.23	0.70	↓**
29	Lyso-PC/17:2-5OH	17	1.07	0.79	↓**	1.22	1.24	↑**
30	24:5/Lyso-PC	24	1.46	2.16	↑**	1.34	0.53	↓**
31	18:1/Lyso-PE	18	1.49	2.46	↑**	1.43	0.41	↓**
32	20:5/Lyso-PE	20	1.10	1.32	↑*	1.47	0.45	↓**
33	Lyso-PE/22:7	22	1.03	0.47	↓*	1.42	2.29	↑**
34	22:5/Lyso-PE	22	1.27	1.50	↑**	1.47	0.32	↓**
35	Lyso-PE/22:4	22	1.12	1.50	↑**	1.39	1.69	↑**
36	5OH-24:5/Lyso-PS	24	1.29	1.74	↑**	1.29	0.53	↓**

Notes: ^#^, isomer; *, *p* < 0.05; **, *p* < 0.01; ^a^, the number of C atoms in the fatty acyl group.

Abbreviations: FC, fold change; VIP, variable importance in the projection.

## Discussion

Hyperlipidemia, a critical risk factor for cardiovascular and cerebrovascular diseases, is characterized in TCM theory by pathological patterns such as *phlegm*, *dampness*, and *blood stasis* (Yan et al. 2022). According to TCM theory, excessive intake of fatty and greasy foods impairs spleen function, leading to the internal accumulation of phlegm and dampness. Therefore, the continuous high-fat diet not only induces biomedical hyperlipidemia but also mimics, to some extent, the etiology and pathological characteristics of phlegm-dampness syndrome in TCM (Gao et al. 2015). ZXD exerts therapeutic effects by eliminating phlegm and dampness, unclogging the liver, and strengthening the spleen, which is consistent with the pathogenesis of hyperlipidemia (Shi et al. 2023; Su et al. 2025). Although the therapeutic efficacy of ZXD in treating hyperlipidemia has been demonstrated, its material basis and mechanism of action remain unclear. In the present study, the lipid-lowering efficacy of ZXD has been demonstrated in a hyperlipidemia mice model. The pharmacodynamic results showed ZXD could significantly improve dyslipidemia and attenuate hepatic lipid accumulation.

Serum pharmacochemistry results showed that 12 compounds entered the bloodstream and underwent metabolic processes after oral administration of ZXD. These constituents are mainly classified into three categories: triterpenoids, sesquiterpenoids and phenolic acids. A study using spectrum-effect analysis demonstrated that alisol B and alisol C 23-acetate were the major lipid-lowering compounds in ZXD (Chang et al. 2022). Alisol B can alleviate hepatocyte lipid accumulation, lipotoxicity, and murine nonalcoholic steatohepatitis *via* the RARα-PPARγ-CD36 regulatory cascade (Zhao et al. 2022). As the parent component of the metabolite 11-demethylation-atractylenolide II, atractylenolide II has been demonstrated to ameliorate hyperlipidemia in mice by regulating the AMPK/PPARα/SREBP-1C signaling pathway (Ren et al. 2019). Several compounds from *Alismatis Rhizoma* and *Atractylodis Macrocephalae Rhizoma* have been demonstrated to exert lipid-lowering effects and are closely associated with the PPAR signaling pathway, suggesting a synergistic effect of the two herbs in the treatment of hyperlipidemia.

Network pharmacology was adopted to elucidate the lipid-lowering mechanism of ZXD in the treatment of hyperlipidemia from a systems perspective and the molecular level. Ten hub targets were recognized through network topology analysis in the PPI network, including PPARG, PPARA, ALB, AKT1, PTGS2, CASP3, ESR1, EGFR, IGF1 and HSP90AA1, indicating that ZXD exerts its therapeutic effects through the coordinated regulation of multiple targets. Analysis of the C-T-D network further highlighted alisol B as a key active constituent of ZXD, consistent with its previously reported lipid-lowering effect (Gao et al. 2024). The results of GO and KEGG pathway analysis revealed that the core molecular mechanism of ZXD against hyperlipidemia may revolve around the PPAR signaling pathway and exert effects through multi-dimensional regulation. At the BP level, there is a significant enrichment of the lipid metabolic process, which is highly consistent with the core function of the PPAR signaling pathway. PPAR family members, key regulators of lipid metabolism, can regulate fatty acid oxidation, fat cell development, lipoprotein metabolism and glucose homeostasis (Li and Glass 2004). At the CC level, the nucleus and cytoplasm were significantly enriched. Targeting the PPARα nucleus-cytoplasm shuttling machinery represents a therapeutic strategy for nonalcoholic fatty liver disease (Zhong et al. 2023). At the MF level, protein binding, DNA binding and nuclear receptor activity were significantly enriched. PPARs form a heterodimer with the Retinoid X Receptor (RXR) prior to binding to DNA, and this process serves as a molecular bridge linking lipid signaling and gene expression (Todisco et al. 2022). These findings indicate that the PPAR signaling pathway is closely associated with the lipid-lowering mechanism of ZXD.

A highly relevant study (Ju et al. 2024) using a high-fat diet-induced hyperlipidemia mouse model demonstrated that ZXD exerts lipid-lowering effects by modulating the miR21/PI3K-Akt/SREBP pathway and improving cholesterol transport. Their findings provide crucial insights at the molecular signaling level. In contrast, the present study offers a complementary perspective from the aspects of *in vivo* absorption constituents and lipid metabolism. Based on serum pharmacochemistry, 12 active constituents directly absorbed into the bloodstream were identified, thereby linking the pharmacodynamic material basis of ZXD with its lipid-lowering effects. Furthermore, network pharmacology and targeted lipidomics analysis indicated that regulation of the PPAR signaling pathway and lysophospholipid metabolism may represent an important mechanism underlying the antihyperlipidemic effect of ZXD. Given the well-documented molecular cross-talk between the PI3K/Akt cascade and PPAR activation in lipid homeostasis (Li et al. 2019), these collective findings strongly suggest that ZXD may act through an integrated, multi-dimensional regulatory network involving PI3K/Akt signaling, PPAR activation, and lysophospholipid regulation.

Lysophospholipids can act as endogenous agonists or antagonists of PPARs, regulate their transcriptional activity by binding to PPARs, and thereby participate in lipid metabolism, inflammation inhibition, cell function regulation, and related diseases (Tsukahara 2013; Klingler et al. 2016; Tsukahara et al. 2017). Accumulating evidence indicates that lysophospholipids are significantly associated with the pathogenesis of hyperlipidemia (Shi et al. 2019; Zhou et al. 2022). Based on these observations, modulation of lysophospholipid metabolism may represent an important mechanism through which the PPAR signaling pathway is regulated in hyperlipidemia. In this context, targeted lipidomics was employed to evaluate the dynamic changes in lysophospholipids under endogenous dyslipidemic conditions and following exogenous intervention with ZXD, thereby enabling a systematic elucidation of the overall antihyperlipidemic mechanism of ZXD. In the study, a total of 36 overlapping differentially lysophospholipids were identified, 33 of which were significantly reversed after ZXD treatment in hyperlipidemia mice. Among the identified lysophospholipids, Lyso-PC (22:4) has been reported to act as a potent agonist of PPARA (Zhang et al. 2022). Lyso-PC (18:2) was found to significantly upregulate the transcriptional and protein expression of PPARA and activate the PPARA-mediated hepatic fatty acid oxidation pathway (He et al. 2026). In addition, Lyso-PC (18:0) and Lyso-PC (18:1) have been reported as potential PPARG agonists (Wang et al. 2020). Lyso-PE (18:1) is also a core intermediate in the biosynthesis of oleoylethanolamine, which binds to PPARA and PPARG as central regulators of lipid and glucose homeostasis and inhibitors of feeding behavior (Ha et al. 2012). These studies suggest that Lyso-PC (22:4), Lyso-PC (18:2), Lyso-PC (18:0), Lyso-PC (18:1), and Lyso-PE (18:1) may represent specific lysophospholipid biomarkers most closely associated with PPARA and PPARG regulation following ZXD treatment. Notably, the number of C atoms in the fatty acyl groups of 36 differential lysophospholipids was predominantly 18 (6 species), 20 (12 species) and 22 (6 species), indicating that lysophospholipids regulated by ZXD primarily feature even number of C atoms in fatty acyl chains. This distribution pattern may be attributed to the physiological preference of animals for synthesizing and storing lipids with even-numbered fatty acid chain (Pinch et al. 2021). Moreover, a study showed that the fatty acid with 22 carbons may have a critical effect on the development or pathogenesis of hyperlipidemia (Shi et al. 2019). Collectively, these findings suggest that the therapeutic effects of ZXD against hyperlipidemia may be closely associated with the regulation of long-chain lysophospholipids possessing even-numbered fatty acyl groups.

Among the identified lysophospholipid subclasses, Lyso-PCs, which are major components of oxidized low-density lipoproteins (oxLDLs), have been reported to play a role in regulating cell proliferation and inflammatory responses (Bellot et al. 2023). A previous study showed that polyunsaturated Lyso-PC (20:4) and Lyso-PC (20:6) are potential inhibitors of the inflammatory effects induced by saturated Lyso-PCs (Timm et al. 2023). In hyperlipidemia mice, a decreased level of 18:0/Lyso-PC may indicate the synthesis of lysophosphatidic acid and arachidonic acid (Mengxiang et al. 2024). The results of this study indicated that ZXD may ameliorate the low-grade inflammation associated with hyperlipidemia by improving the metabolism of Lyso-PC. In addition, Lyso-PEs have been implicated in hepatic lipid accumulation and metabolism, potentially inducing cellular lipid droplet formation and altering triacylglycerol profiles (Yamamoto et al. 2022). By decreasing levels of 18:1/Lyso-PE, 22:5/Lyso-PE and 20:5/Lyso-PE, ZXD may inhibit fatty liver formation by reducing hepatic lipid accumulation, thereby contributing to its overall lipid-lowering and hepatoprotective effects.

## Conclusions

This study provided significant insights into the pharmacodynamics material basis of ZXD in the treatment of hyperlipidemia and elucidated its potential molecular mechanism by integrating serum pharmacochemistry, network pharmacology, and targeted lipidomics. A total of 12 compounds absorbed into the bloodstream after oral administration of ZXD were identified as potential active constituents. The PPAR signaling pathway involved in the antihyperlipidemic effects of ZXD, which may be closely associated with ZXD-mediated modulation of lysophospholipid metabolism. Despite these findings, several limitations should be acknowledged. First, this study was based on a single animal model, and the generalizability of the findings requires further validation in additional models. Second, although the integrated analysis suggested a close association between the identified lysophospholipid biomarkers and PPAR activation, direct experimental evidence confirming their causal relationship remains lacking. Future studies are therefore needed to further verify these molecular mechanisms and to clarify the potential synergistic effects of the active constituents of ZXD. Collectively, these findings not only deepen the understanding of the material basis and mechanisms of action of ZXD against hyperlipidemia but also provide a novel and integrative paradigm for investigating the ­pharmacological effects of TCM prescriptions.

## Supplementary Material

Supplementary Material revised.docx

ARRIVE guidelines.pdf

## Data Availability

The data that support the findings of this study are available from the corresponding author, [Pan Yan], upon reasonable request.
