# Supplementary material for: Zexie decoction alleviates hyperlipidemia through modulation of the PPAR signaling pathway and lysophospholipid metabolism
Source: Pharm Biol. 2026 Jun 15;64(1):848–65. doi: 10.1080/13880209.2026.2687990 (PMC13270863; doi:10.1080/13880209.2026.2687990)
Supplement: Supplementary Material revised.docx [file IPHB_A_2687990_SM4612.docx]

**Supplementary Material**

**Figure S1**. Baseline levels of plasma biochemical indicators in each group before drug administration. (A) TC, (B) TG, (C) HDL-C, (D) LDL-C. ^#^*P* < 0.05, ^##^*P* < 0.01 versus NC group.

**Figure S2**. The BPC of ZXD extracts detected by UHPLC-QTOF-MS/MS.

(A) Negative ion mode, (B) Positive ion mode.

**Figure S3**. The BPC of serum sample detected by UHPLC-QTOF-MS/MS.

(A) Negative ion mode of dosed serum sample, (B) Negative ion mode of blank serum sample, (C) Positive ion mode of dosed serum sample, (D) Positive ion mode of blank serum sample.


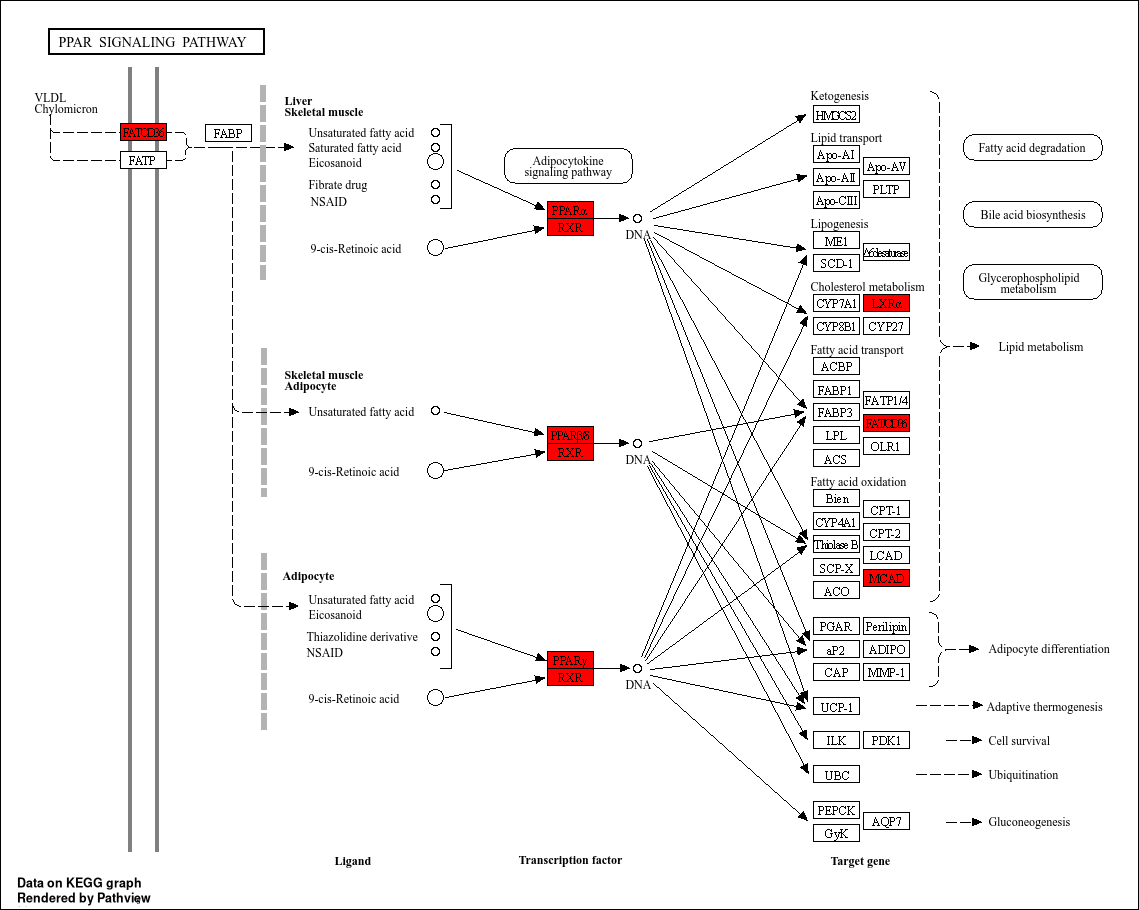


**Figure S4.** The effect of PPAR signaling pathway in the treatment of hyperlipidemia by ZXD. Red block diagrams represent genes that are enriched in the pathway.

**Figure S5**. Redocking of the co-crystallized ligands T4A and EWR into PPARG and PPARA, respectively. (A) PPARG-T4A complex, (B) PPARA-EWR complex.

**Table S1.** Identification of chemical compounds in ZXD extracts by UHPLC-MS/MS.

| **No.** | **RT (min)** | **Adduct** | **Experimental**  **(m/z)** | **Calculated**  **(m/z)** | **Error**  **(ppm)** | **Formula** | **MS/MS Fragments** | **Identification** |
| --- | --- | --- | --- | --- | --- | --- | --- | --- |
| 1 | 1.94 | [M-H]^-^ | 191.0204 | 191.0197 | 3.7 | C_6_H_8_O_7_ | 191.0181, 111.0082, 87.0081, 85.0290, 57.0342 | Citric acid |
| 2 | 3.58 | [M+FA-H]^-^ | 873.2765 | 873.2729 | 4.1 | C_30_H_52_O_26_ | 827.2710, 665.2193, 503.1648, 341.1084, 179.0559 | Maltopentaose |
| 3 | 4.68 | [M+H]^+^ | 289.1399 | 289.1394 | 1.7 | C_12_H_20_N_2_O_6_ | 289.1385, 271.1270, 253.1179, 235.1071, 217.0958, 175.0853, 163.0857, 147.0905 | 4,4′-(2,6-Pyrazinediyl)bis[1,2,3-butanetriol] |
| 4 | 5.51 | [M+H]^+^ | 268.1034 | 268.1040 | -2.2 | C_10_H_13_N_5_O_4_ | 136.0617, 119.0349 | Adenosine |
| 5 | 6.14 | [M-H]^-^ | 282.0828 | 282.0839 | -3.9 | C_10_H_13_N_5_O_5_ | 282.0847, 150.0415, 133.0150, 108.0192 | Guanosine |
| 6 | 6.46 | [M+H]^+^ | 283.1405 | 283.1401 | 1.4 | C_12_H_18_N_4_O_4_ | 265.1284, 178.0852, 162.0917, 134.0962 | N2-[2-(2-Furanyl)-2-oxoethyl]-L-arginine |
| 7 | 7.823 | [M+FA-2H]^2-^ | 864.2727 | 864.2671 | 6.5 | C_61_H_104_O_53_ | 841.2669, 818.2633, 241.1089, 179.0566 | O-β-D-galactopyranosyl-(1→7)-O-D-glycero-α-D-manno-heptopyranosyl-(1→6)-O-D-glycero-α-D-manno-heptopyranosyl-(1→6)-O-β-D-glucopyranosyl-(1→4)-O-[O-L-glycero-α-D-manno-heptopyranosyl-(1→2)-L-glycero-α-D-manno-heptopyranosyl-(1→3)]-O-[α-D-glucopyranosyl-(1→6)]-O-L-glycero-α-D-manno-heptopyranosyl-(1→5)-3-deoxy-α-D-manno-2-Octulopyranosonic aci*d* |
| 8 | 10.98 | [M-H]^-^ | 203.0829 | 203.0826 | 1.5 | C_11_H_12_N_2_O_2_ | 203.0829, 159.0939, 142.0678, 116.0500, 74.0245 | L-Tryptophan |
| 9 | 11.57 | [M+H]^+^ | 297.1553 | 297.1557 | -1.3 | C_13_H_20_N_4_O_4_ | 297.0942, 279.0874, 219.0678, 192.0622, 159.0594, 132.0539, 117.0339 | 2-[1-Ethyl-3-(hydroxyimino)-3-(2-hydroxy-5-methoxyphenyl)propyl]hydrazinecarboxamide |
| 10 | 11.70 | [M-H]^-^ | 253.1193 | 253.1194 | -0.4 | C_12_H_18_N_2_O_4_ | 253.1184, 223.1096, 124.0398, 94.0296 | Methyl 4-[[(1,1-dimethylethoxy)carbonyl]amino]-1-methyl-1H-pyrrole-2-carboxylate |
| 11 | 11.95 | [M-H]^-^ | 337.1151 | 337.1135 | 4.7 | C_13_H_22_O_10_ | 337.1121, 235.0822, 193.0726, 125.0238 | 6-O-Methylagarobiose |
| 12 | 14.64 | [M-H]^-^ | 353.0868 | 353.0878 | -2.8 | C_16_H_18_O_9_ | 353.0887, 191.0567, 179.0356, 135.0457 | Neochlorogenic acid |
| 13 | 15.61 | [M-H]^-^ | 351.1317 | 351.1291 | 7.4 | C_14_H_24_O_10_ | 351.1395, 207.0888, 161.0458, 125.0251, 99.0460, 57.0353 | (1R)-2-Carboxy-1-methylethyl (3R)-3-(D-glucopyranosyloxy)butanoate |
| 14 | 16.26 | [M+H]^+^ | 295.1647 | 295.1652 | -1.7 | C_15_H_22_N_2_O_4_ | 177.0542, 145.0280, 117.0330, 89.0380 | N-(4-Aminobutyl)-3-(4-hydroxy-3,5-dimethoxyphenyl)-2-propenamide |
| 15 | 16.41 | [M+H]^+^ | 265.1551 | 265.1547 | 1.5 | C_14_H_20_N_2_O_3_ | 177.0549, 145.0287, 117.0336, 89.0381 | Feruloylputrescine |
| 16 | 17.35 | [M+FA-H]^-^ | 493.2309 | 493.23 | 1.8 | C_21_H_36_O_10_ | 493.2298, 447.2229, 285.1688, 119.0333 | Atractyloside A |
| 17 | 17.85 | [M+H]^+^ | 350.1825 | 350.1823 | 0.6 | C_16_H_23_N_5_O_4_ | 350.1804, 291.1318, 205.0960, 160.0747, 132.0795, 98.0594 | N-Benzoyl-L-alanyl-L-arginine |
| 18 | 17.93 | [M+H]^+^ | 279.1704 | 279.1703 | 0.4 | C_15_H_22_N_2_O_3_ | 177.0537, 145.0275, 117.0328, 89.0375 | Feruloylcadaverine |
| 19 | 18.14 | [M-H]^-^ | 353.0873 | 353.0878 | -1.4 | C_16_H_18_O_9_ | 353.0908, 191.0566, 179.0357, 173.0460, 135.0445 | Chlorogenic acid |
| 20 | 18.37 | [M-H]^-^ | 210.0773 | 210.0772 | 0.5 | C_10_H_13_NO_4_ | 124.0408, 106.0305, 94.0306, 66.0352 | 3-Methoxy-L-tyrosine |
| 21 | 18.47 | [M-H]^-^ | 353.0864 | 353.0878 | -4.0 | C_16_H_18_O_9_ | 191.0550, 173.0451, 85.0292 | Cryptochlorogenic acid |
| 22 | 18.67 | [M-H]^-^ | 325.0571 | 325.0565 | 1.8 | C_14_H_14_O_9_ | 193.0508, 178.0258, 149.0603, 134.0371 | trans-Fertaric acid |
| 23 | 18.69 | [M+H]^+^ | 428.1928 | 428.1928 | 0.0 | C_21_H_25_N_5_O_5_ | 428.1918, 369.1435, 205.0964, 163.0490 | N-(Aminoiminomethyl)-L-tyrosyl-L-phenylalanylglycine |
| 24 | 19.02 | [M+FA-H]^-^ | 477.2345 | 477.2341 | 0.8 | C_21_H_36_O_9_ | 477.2347, 431.2294, 269.1761, 161.0449 | (1S,3aS,4R,7R,8aR)-7-[1-(β-D-Glucopyranosyloxy)-1-methylethyl]octahydro-4-hydroxy-4-(hydroxymethyl)-1-methyl-2(1H)-azulenone |
| 25 | 19.92 | [M+H]^+^ | 412.1967 | 412.1979 | -2.9 | C_21_H_25_N_5_O_4_ | 412.1981, 395.1711, 353.1501, 307.1440 | N-(Aminoiminomethyl)-L-phenylalanyl-L-phenylalanylglycine |
| 26 | 21.33 | [M+H]^+^ | 471.2113 | 471.2126 | -2.8 | C_25_H_30_N_2_O_7_ | 295.1635, 277.1533, 248.1273, 177.0536, 145.0272 | N,N′-cis-diferuloyl-3-hydroxy-cadaverine |
| 27 | 21.50 | [M+H]^+^ | 389.2524 | 389.2547 | -5.9 | C_21_H_32_N_4_O_3_ | 389.2524, 330.2062, 311.2111, 244.1682, 232.1695 | N-[4-[(Aminoiminomethyl)amino]butyl]-N-[(2,3-dihydro-1,4-benzodioxin-6-yl)methyl]cyclohexanecarboxamide |
| 28 | 21.99 | [M+H]^+^ | 441.2009 | 441.202 | -2.5 | C_24_H_28_N_2_O_6_ | 441.2038, 265.1539, 177.0504, 145.0279 | N,N′-Diferuloylputrescine |
| 29 | 22.38 | [M+H]^+^ | 455.2165 | 455.2177 | -2.6 | C_25_H_30_N_2_O_6_ | 279.1688, 177.0540, 145.0281 | Diferulylcadaverine |
| 30 | 22.41 | [M+H]^+^ | 309.0868 | 309.0870 | -0.6 | C_17_H_12_N_2_O_4_ | 291.0743, 263.0794, 206.0823, 180.0793 | Flazine |
| 31 | 22.61 | [M-H_2_O+H]^+^ | 203.1799 | 203.1794 | 2.5 | C_15_H_24_O | 203.1799, 161.1327, 147.1170, 133.1013, 119.0850, 105.0690 | (-)-Spathulenol |
| 32 | 23.05 | [M-H]^-^ | 327.2175 | 327.2177 | -0.6 | C_18_H_32_O_5_ | 327.2163, 291.1966, 229.1435, 211.1328, 171.1017 | 9,12,13-Trihydroxy-10,15-octadecadienoic acid |
| 33 | 23.54 | [M-H]^-^ | 329.2333 | 329.2328 | 1.5 | C_18_H_34_O_5_ | 329.2333, 311.2213, 201.1125, 171.1021 | 9,10,13-Trihydroxy-(E)-11-octadecenoic acid |
| 34 | 24.03 | [M+FA-H]^-^ | 549.3444 | 549.3433 | 2.0 | C_30_H_48_O_6_ | 549.3402, 485.3292, 367.2646 | 16-Oxoalisol A |
| 35 | 24.36 | [M+FA-H]^-^ | 591.3555 | 591.3539 | 2.7 | C_32_H_50_O_7_ | 591.3557, 545.3514, 527.3394, 485.3300, 467.3181, 367.2633 | 16-oxoalisol A 23-acetate |
| 36 | 24.89 | [M+FA-H]^-^ | 591.3563 | 591.3539 | 4.1 | C_32_H_50_O_7_ | 591.3350, 545.3459, 527.3374, 467.3144 | 16-oxoalisol A 24-acetate |
| 37 | 24.89 | [M+FA-H]^-^ | 531.3340 | 531.3327 | 2.4 | C_30_H_46_O_5_ | 485.3291, 467.3199, 367.2622 | 16-Oxo-11-anhydroalisol A |
| 38 | 25.27 | [M+FA-H]^-^ | 551.3608 | 551.3589 | 3.4 | C_30_H_50_O_6_ | 551.3596, 505.3524, 271.1926, 223.1548 | 13,17-Epoxyalisol A |
| 39 | 25.29 | [M+FA-H]^-^ | 531.3315 | 531.3327 | -2.3 | C_30_H_46_O_5_ | 531.3380, 485.3316, 467.3202, 409.2773 | Alisol C |
| 40 | 25.65 | [M-H_2_O+H]^+^ | 231.1373 | 231.1380 | -3.0 | C_15_H_20_O_3_ | 231.1367, 213.1281, 189.0905, 163.0738, 105.0697 | Atractylenolide Ⅲ |
| 41 | 25.75 | [M+FA-H]^-^ | 531.3326 | 531.3327 | -0.2 | C_30_H_46_O_5_ | 531.3396, 485.3305, 367.2644 | (8α,9β,11β,12β,14β,24S)-24,25-Epoxy-11,12-dihydroxydammar-13(17)-ene-3,23-dione |
| 42 | 25.94 | [M-H]^-^ | 281.1391 | 281.1394 | -1.1 | C_15_H_22_O_5_ | 281.1382, 219.1389, 193.1230, 191.1436 | (4aR,8aR,9aS)-Decahydro-4,6,9a-trihydroxy-3,8a-dimethyl-5-methylenenaphtho[2,3-b]furan-2(3H)-one |
| 43 | 26.91 | [M+FA-H]^-^ | 573.3440 | 573.3433 | 1.2 | C_32_H_48_O_6_ | 573.3498, 527.3441, 509.3328, 467.3224, 449.3109, 391.2676 | Alisol C 23-acetate |
| 44 | 27.34 | [M+H]^+^ | 233.1536 | 233.1536 | 0.0 | C_15_H_20_O_2_ | 233.1524, 215.1363, 187.1478, 159.0810, 131.0863 | Atractylenolide Ⅱ |
| 45 | 27.81 | [M+FA-H]^-^ | 535.3674 | 535.3640 | 6.4 | C_30_H_50_O_5_ | 535.3618, 471.3493, 339.2597 | Alisol A |
| 46 | 28.07 | [M+FA-H]^-^ | 577.3721 | 577.3746 | -4.3 | C_32_H_52_O_6_ | 577.3731, 531.3678 | Alisol A 24-acetate |
| 47 | 28.505 | [M+H]^+^ | 231.1375 | 231.1380 | -2.2 | C_15_H_18_O_2_ | 231.1372, 185.1328, 157.1006, 128.0617 | Atractylenolide Ⅰ |
| 48 | 28.86 | [M+FA-H]^-^ | 577.3772 | 577.3746 | 4.5 | C_32_H_52_O_6_ | 577.3749, 531.3676 | Alisol A 23-acetate |
| 49 | 29.65 | [M-H_2_O+H]^+^ | 455.3508 | 455.352 | -2.6 | C_30_H_48_O_4_ | 455.3507, 437.3383, 419.3309, 383.2917, 339.2667 | Alisol G |
| 50 | 29.98 | [M-H_2_O+H]^+^ | 455.3513 | 455.3520 | -1.5 | C_30_H_48_O_4_ | 455.3517, 437.3416, 419.3308, 383.2941, 365.2831, 339.2670 | Alisol B |
| 51 | 31.30 | [M+H]^+^ | 515.3745 | 515.3731 | 2.7 | C_32_H_50_O_5_ | 515.3752, 479.3502, 437.3447, 419.3268, 365.2852, 357.2793 | Alisol B 23-acetate |
| 52 | 33.40 | [M+H]^+^ | 495.3482 | 495.3469 | 2.6 | C_32_H_46_O_4_ | 495.3516, 417.3197, 363.2694, 183.1154 | 11,25-Anhydroalisol F 24-acetate |
| 53 | 34.01 | [M+H]^+^ | 499.3769 | 499.3782 | -2.6 | C_32_H_50_O_4_ | 499.3769, 439.3585, 341.2830, 215.1802 | 11-deoxyalisol B 23-acetate |

**Table S2.** The hub targets screened from PPI network by CytoHubba.

| **Gene symbol** | **MCC** | **MNC** | **Degree** | **EPC** | **BottleNeck** | **EcCentricity** | **Closeness** | **Radiality** | **Betweenness** | **Stress** |
| --- | --- | --- | --- | --- | --- | --- | --- | --- | --- | --- |
| ALB | 6.975068274E9 | 44 | 44 | 33.052 | 2 | 0.25 | 55.08333 | 4.70149 | 519.54777 | 2404 |
| PPARG | 6.970683051E9 | 42 | 43 | 33.166 | 6 | 0.33333 | 54.83333 | 4.71642 | 637.23226 | 3006 |
| AKT1 | 6.975096854E9 | 43 | 43 | 33.494 | 7 | 0.25 | 54.41667 | 4.67164 | 398.95578 | 2424 |
| PTGS2 | 6.894885774E9 | 36 | 36 | 32.556 | 5 | 0.25 | 50.75 | 4.55224 | 214.18014 | 1418 |
| ESR1 | 6.9326022E9 | 34 | 34 | 31.746 | 4 | 0.25 | 49.75 | 4.52239 | 150.34275 | 1182 |
| IGF1 | 6.967481994E9 | 32 | 32 | 31.786 | 7 | 0.25 | 48.75 | 4.49254 | 112.97086 | 960 |
| PPARA | 1.23039186E8 | 29 | 29 | 30.156 | 8 | 0.33333 | 47.5 | 4.47761 | 191.41251 | 1344 |
| CASP3 | 6.97504272E9 | 29 | 29 | 31.491 | 1 | 0.25 | 47.25 | 4.44776 | 47.51194 | 472 |
| EGFR | 6.968799816E9 | 29 | 29 | 31.15 | 1 | 0.25 | 47.25 | 4.44776 | 91.00961 | 850 |
| HSP90AA1 | 6.9535604E9 | 28 | 28 | 30.887 | 2 | 0.25 | 46.75 | 4.43284 | 104.54779 | 810 |

**Table S3.** Docking scores of the absorbed compounds and metabolites with PPARA and PPARG.

| **Compound** | **PPARA (kcal/mol)** | **PPARG (kcal/mol)** |
| --- | --- | --- |
| Atractyloside A | -8.0 | -7.5 |
| Alisol C 23-acetate | -5.5 | -7.6 |
| Alisol B | -5.4 | 7.3 |
| 11-demethylation-atractylenolide Ⅱ | -8.5 | -6.6 |
| 16-hydroxy-alisol B | -5.6 | -7.7 |
